# Thoracic ultrasound alone or in combination with tracheal amylase as a tool predictor of ventilator-associated pneumonia in neurocritical patients

**DOI:** 10.1097/MD.0000000000032149

**Published:** 2022-12-02

**Authors:** Roberto Mercado-Longoria, Juan O. Galindo-Galindo, Mario A. Ataxca-Gonzalez, Perla R. Colunga-Pedraza, Samantha P. Peña-Lozano, Jorge M. Llaca-Díaz, Erick J. Rendón-Ramírez

**Affiliations:** a Pulmonary and Critical Care Medicine Service, Hospital Universitario Dr. José Eleuterio González, Universidad Autónoma de Nuevo León, Monterrey, México; b Internal Medicine, Department, Hospital Universitario Dr. José Eleuterio González, Universidad Autonoma de Nuevo León, Monterrey, México; c Clinical Pathology Department, Hospital Universitario, UANL, Monterrey, Nuevo León, México.

**Keywords:** Amylase, neurosurgery, thoracic ultrasound, traumatic brain injury, ventilator-associated pneumonia

## Abstract

In this study, we aim to evaluate whether thoracic ultrasound (TUS) and tracheal amylase (TA) alone or in combination can predict the development of ventilator-associated pneumonia (VAP) in neurocritical patients. Consecutive adult patients with neurocritical disease with normal chest radiographs who required intensive care unit admission and mechanical ventilation between March 2015 and July 2018 were included. TUS and Amylase levels were measured during the first 24 hours and repeated 48 hours after orotracheal intubation. Forty-three patients with a median age of 34 years (17–82) were included. TUS had a sensitivity of 100% and specificity of 96.3% as a predictor of VAP within the first 48 hours when nonpattern A was observed. TA levels > 200 UI/L in the first 48 hours had a sensitivity of 87.5%, and specificity of 63% as a predictor of VAP. Moreover, no benefit of TUS plus TA compared to TUS alone as a predictor of VAP was found. The identification of abnormal TUS patterns in the first 48 hours of orotracheal intubation is a significant predictor of VAP in neurocritical patients.

## 1. Introduction

The incidence of ventilator-associated pneumonia (VAP) in neurocritical patients remains high (50%), with an associated mortality of 20 to 50%. In addition, previous studies have demonstrated that patients diagnosed with VAP have an increased length of intensive care unit (ICU) stay and related healthcare costs.^[1–3]^

VAP is a nosocomial infection that affects patients who receive mechanical ventilation (MV) for > 48 hours. A combination of clinical criteria, such as the Clinical Pulmonary Infection Score (CPIS), has been proposed for the diagnosis of VAP using X-rays as the imaging modality. However, CPIS has shown poor diagnostic performance.^[4–8]^

Risk factors for VAP, such as microaspiration of oropharyngeal secretions or gastric contents in neurocritical patients, are key to its pathogenesis.^[4]^

Currently, there are no validated biomarkers for VAP diagnosis. Several biomarkers have been studied to predict the severity and prognosis of VAP, such as interleukin-18 (IL-18) and soluble triggering receptor expressed on myeloid cells 1 (sTREM-1),^[9,10]^ histidine-rich glycoprotein,^[11]^ pentraxin 3,^[12]^ procalcitonin,^[13,14]^ breath octane, acetaldehyde,^[15]^ and C-reactive protein.^[16]^ Procalcitonin, C-reactive protein, sTREM-1, and HRC have proven to be predictors of VAP; however, most are markers of inflammation, making them nonspecific.^[9–11,13,14,16]^

Recently, the α-amylase concentration in tracheal secretions and bronchoalveolar lavage has been reported as a potentially useful marker for diagnosing microaspiration and bacterial pneumonia.^[3,5,17]^ This marker is inexpensive and easily measured and could be an attractive marker for predicting the development of early VAP.^[18]^

In the last decade, there has been an increase in thoracic ultrasound (TUS) evaluation in the diagnosis of VAP with almost 100% sensitivity,^[19]^ and few studies have proven the utility of TUS as a tool predictor in detecting lung changes prior to VAP diagnosis.^[20]^

Currently, there are no clinical scores or biomarkers to predict the occurrence of VAP.^[21]^

This study aimed to evaluate whether TUS alone or in combination with tracheal amylase (TA) is a significant predictor of VAP in patients with neurocritical disease.

## 2. Methods

The study was approved by our ethics committee on January 28, 2016 (NM16-00001) and was conducted in accordance with the Declaration of Helsinki. All the patients provided informed consent to participate in the study.

### 2.1. Study subjects

We prospectively recruited patients aged > 18 years with neurocritical diseases who were admitted to the ICU, between January 2016 and July 2018, were on MV for < 48 hours in order to protect the upper airway, and presented with normal chest X-ray. Patients suspected of having a respiratory tract infection, chronic infections (hepatitis B and C, human immunodeficiency virus), chronic use of steroids, neurogenic pulmonary edema, or pulmonary contusions (thoracic trauma and chest X-ray infiltration) were excluded. All the patients were treated using a standard ICU protocol to prevent pneumonia.

### 2.2. Data collection

All patients were followed up for the next 30 days to determine who developed VAP with the Centers for Disease Control and Prevention (CDC) and CPIS.^[6,7]^ VAP was defined as a pulmonary infection that developed in-hospital care 48 hours after intubation and MV. VAP was diagnosed by an Intensive Care medical team composed of Internal Medicine, Pulmonology, and Critical Care, using the major CDC criteria or more than 6 points in the CPIS criteria. The Intensive Care medical team carried out its VAP diagnostic approach blinded to the TUS results.

Amylase levels were measured 24 and 48 hours after intubation using an enzymatic method (Beckman Coulter, CA).^[19]^ Tracheal suctioning was routinely performed using an open tracheal suction system with a 14 French endotracheal tube and 10 mL of sterile normal saline instilled into the trachea before endotracheal suctioning.

TUS was performed in a semi-upright position during the first 24 hours and repeated 48 hours after orotracheal intubation using a Z6 ultrasound equipment (Mindray equipment, Nanshan, Shenzhen, China) with a low-frequency convex transducer (5 Hz).

Four points per hemithorax (anterior and lateral chest walls, with upper and lower areas in each zone) were evaluated.^[22]^ The thoracic posterior segments were not assessed because most patients could not adopt a lateral or sitting position. Only one pulmonologist with 6 years of experience in TUS, was blinded to the clinical data, and follow-up was performed on all TUS.

The following ultrasound parameters were recorded as pulmonary findings: normal lung pattern (lung sliding and A lines), abnormal TUS pattern (B lines, Subpleural space, fractal sign, dynamic or static air bronchogram, tissue-like, shred sign, and pleural effusion).^[22]^

To prevent VAP clinical staff followed the guideline recommendations, such as keeping the patient’s bed in a semifowler position (between 30 and 45º), taking off the ventilator once the patient no longer required it, and maintaining good hygiene.^[4]^

Other variables analyzed were hospitalization days, difficult orotracheal intubation according to the American Studies Association Task Force, and severity of neurocritical disease using the Glasgow score, Sequential Organ Failure Assessment Score (SOFA) score, and Acute Physiology and Chronic Health Disease Classification System (APACHE) II score.

### 2.3. Statistical analysis

In descriptive statistics, general and specific variables were analyzed, obtaining measures of central tendency and dispersion, and determining normality with the Kolmogorov–Smirnov test.

In inferential statistics, categorical variables were analyzed using Pearson’s Chi-square test. Statistical significance was set at *P* < .05. Statistical analyses were performed using SPSS Software (IBM Corp. Released in 2011. IBM SPSS Statistics for Windows, Version 20.0. software IBM Corp Armonk, New York).

## 3. Results

### 3.1. Baseline characteristics

Forty-three patients with neurocritical disease met the eligibility criteria. Their median age was 34 years (17–82), and 35 were male (81.4%). The most common neurocritical diagnoses were subdural hematoma in ten patients (23.25%), intraparenchymal hemorrhage in 9 patients (20.93%), and subarachnoid hemorrhage in 7 patients (16.25%). Twenty-eight patients (65.1%) required decompressive craniectomy, and there was no significant difference between patients who developed VAP and those who did not. Difficult orotracheal intubation was identified in ten patients (23.3%), with no increase in the development of VAP compared to no difficult intubation. Prophylactic antibiotics were administered to 22 patients (51.1%) at the discretion of the neurosurgeon based on the infection risk associated with skull fractures; the most common antibiotic was cephalothin in 21 patients (48.8%).

Gastric protection in all patients with the most common drug, was Omeprazole in 42 patients (97.67%). Emesis or gastric residue > 500 mL was presented in 6 patients (37.5%) who developed VAP versus 2 patients (7.4%) in the non-VAP group, with statistical significance (*P* = .037) (Table 1). The length of stay (LOS) in the ICU had a median of 5 days (0–22 d), the median LOS in the hospital ward was 14 days (3–95 d), and there were no statistical differences in LOS and mortality between patients with and without VAP. The baseline and clinical characteristics of patients are shown in Table 1.

**Table 1:** Demographic and baseline characteristics of the 43 patients.

Characteristics	VAP (n = 16)	No VAP(n = 27)	*P*
Age, median (range)	37.5 (18–82)	33 (17–69)	.74
Gender, n (%)			.688
Male	14 (87.5)	21 (77.8)	
Female	2 (12.5)	6 (22.2)	
ICU median length of stay, d, (range)	8.5 (0–22)	4 (0–19)	.48
Median length of stay, d, (range)	15.5 (4–45)	14 (3–95)	.98
Glasgow, median (range)	7 (3–13)	7 (3–15)	.59
Clinical status, n (%)			<.99
Alive	11 (68.8)	19 (70.4)	
Death	5 (31.3)	8 (29.6)	
BMI, n (%)			.512
> 30	4 (25)	10 (37)	
< 30	12 (75)	17 (63)	
DTI, n (%)	5 (31.3)	5 (18.5)	.46
Craniectomy, n (%)	9 (56.3)	19 (70.4)	.50
Emesis/gastric residue, n (%)	6 (37.5)	2 (7.4)	.037
SOFA, median (range)			
24 h	6 (3–16)	6 (0–14)	.49
48 h	6.5 (4–14)	7 (0–11)	.44
APACHE II admission, median (range)	16.5 (11–28)	17 (2–126)	.54
PAFI, n (%)			.73
> 300	8 (50)	11 (40.7)	
< 300	8 (50)	16 (59.2)	

BMI = body mass index, DTI = difficult tracheal intubation, ICU = intensive care unit, VAP = ventilator-associated pneumonia.

VAP was identified in 16 patients (37.2%) of the total population with neurocritical diseases. The severity of neurocritical disease was evaluated using the Glasgow score, SOFA score, and APACHE II score; and the median Glasgow score in the total population was 7 points (3–15). In 36 patients (83.7%) the Glasgow score was less than 8 at the time of ward admission and required urgent intubation. Glasgow score higher than 13 points were identified in 4 patients (9.3%) with brain tumors and intubation was performed in the surgical room. There was no association between Glasgow score and urgent intubation with the development of VAP (*P* = .59). The median SOFA score in the first 24 hours was 6 points (3–16) in the VAP group versus 6 (0–14) in the non-VAP group, without a statistically significant difference (*P* = .49) and the median SOFA score at 48 hours was 6.5 points (4–14) in the VAP group versus 7 (0–14) in the non-VAP group, without a statistically significant difference (*P* = .44) (See Table 1). The median APACHE II score in the first 24 hours was 16.5 (11–28) in the VAP group versus 17 (2–126) in the non-VAP group, without a statistically significant difference (*P* = .54).

### 3.2. Thoracic ultrasound as a predictor of VAP

Non-A pattern or abnormal TUS (B lines, tissue-like, or other) was identified in the first 24 hours in 7 patients (43.7%) who developed VAP with a sensitivity of 43.8%, specificity of 96.3%, positive predictive value (PPV) of 87.5%, and negative predictive value (NPV) of 74.3%.

Non-A pattern or abnormal TUS (B lines or tissue-like or other in the first 48 hours had a sensitivity of 100%, specificity of 96.3%, PPV of 94.1%, NPV of 100%, and accuracy of 98.15 as predictors of VAP (Tables 2 and 3).

**Table 2:** Ultrasound findings 24 and 48 h after admission to the ICU in patients who developed early VAP (n = 16).

Thoracic ultrasound pattern	<24 h after admission to ICU	<48 h after admission to ICU
A-pattern, n (%)	9 (56.3)	0 (0)
Focal B-lines (A/B), n (%)	4 (25)	12 (75)
Diffuse B-lines, n (%)	2 (12.5)	2 (12.5)
Tissue like n (%)	1 (6.3)	2 (12.5)

ICU = intensive care unit.

**Table 3:** Diagnostic measures of tracheal amylase, thoracic ultrasound, and its combination as predictors of VAP.

	Sensitivity (%)	Specificity (%)	PPV (%)	NPV (%)	Accuracy
TUS at 48 hours	100	96.3	94.1	100	98.15
TA at 48 hours	87.5	63	58.3	89.5	75.25
TUS + TA at 48 hours	100	59.3	59.3	100	79.65

NPV = negative predictive value, PPV = positive predictive value, TA = tracheal amylase, TUS = thoracic ultrasound.

### 3.3. TA as a predictor of VAP

TA levels in the first 24 hours did not predict VAP development (*P* = .24). The TA obtained less than 48 hours had a median of 904 (30–7941) in patients who developed VAP versus non-VAP 111 (0–14,047) with statistical significance (*P* = .022) (Table 4). TA levels > 200 UI/L in the first 48h had a sensitivity of 87.5%, specificity of 63%, PPV of 58.3%, and NPV of 89.5% as predictors of VAP (Table 3).

**Table 4:** Amylase and ventilator-associated pneumonia association.

	VAP	No VAP	*P*
Amylase 24 hours, median (range)	668 (51–57,343)	487 (38–50,647)	.24
Amylase 48 hours, median (range)	904 (30–7941)	111 (0–14,047)	.022

VAP = ventilator-associated pneumonia.

### 3.4. TUS combined with amylase as a predictor of VAP

When amylase was combined with TUS in the first 24 hours identified the sensitivity, specificity, PPV, and NPV were 68.8%, 48.1%, 44%, and 72.2%, respectively. When the TUS and TA tests were evaluated together at 48 hours the sensibility and NPV were 100%, specificity and PPV were 59.3%, and accuracy was 79.65 (Table 3).

## 4. Discussion

In the present study, we found that TUS alone was a better predictor of VAP than TA alone or in combination with TUS.

The inferior prediction of VAP with the combination of TUS and TA compared with TUS alone could be explained by the fact that TA is a good predictor of microaspiration; however, not all patients developed VAP. In contrast, TUS alone shows structural damage (non-A pattern), and thus predicts a more efficient development of VAP. In contrast, Zhou et. al. reported the superiority of TUS and a different biomarker (procalcitonin) combination with a sensitivity and specificity of 81% and 86% respectively, over TUS alone (sensitivity 92%, specificity 63%) for VAP diagnosis, but was not proven to be a predictor of VAP.^[23]^

In our study, TUS alone and in combination showed high sensitivity (100%). Although TUS alone remained highly specific, it was not the same for the combination of TUS and amylase (96.3% vs 59.3%) after 48 hours. In contrast, previous studies on TUS reported low specificity (60%) and high sensitivity (100%),^[19]^ probably because of the high prevalence of atelectasis and the TUS limitation in differentiating between inflammatory and noninflammatory consolidations in these previous studies.

The identification of abnormal TUS patterns (B lines, tissue-like, or others) in the first 48 hours of MV in neurocritical patients predicted the development of VAP before diagnosis using the classic criteria (CPIS or CDC) with a PPV of 94.1%. Moreover, when TUS was normal at 48h of MV (lung sliding, A lines), the NPV was 100%, with a high accuracy of 98.15%.

We already know the great accuracy of point-of-care TUS for the diagnosis of VAP with almost 100% sensitivity^,[19]^ besides it is a great tool requiring less than 5 minutes, no radiation exposure, and it can be performed at the patient’s bed. Although, the overall prediction of VAP using TUS focuses on detecting lung changes before the diagnosis of VAP has been proven but it has been evaluated in a few studies.^[20]^

We evaluated whether other classic risk factors for VAP were associated with its development in addition to abnormal TUS, such as difficult Intubation, time of intubation, points in GCS, and prophylactic antibiotics, but there is no relation with the development of VAP, besides solely emesis or gastric residue > 500ml was associated with the development of VAP (*P* = .037). In addition, prophylactic antibiotics were administered in the surgical room to 22 patients (51.1%) most of whom had traumatic brain injuries at the discretion of the neurosurgeon based on the risk of meningeal infection. Unfortunately, the information about this topic remains controversial; however, some studies are in favor of prophylactic antibiotics, but other studies did not identify any benefit and could be a risk factor for resistant infections.^[24,25]^

The other important factor that we incorporated in our study is TA because according to recent studies, median values of α-amylase in “tracheal” secretions could vary widely from 0 U/L in a small pilot study to over 22,000 U/L in patients with documented aspiration or VAP.^[5]^ Qu et al^[3]^ reported a 90% sensitivity and 79% specificity for identifying VAP with an α-amylase of 4681 U/L; nevertheless, another study showed a contradictory result with a sensitivity of 87% and specificity of 29% for α-amylase (1685 UI/L^−^1) in diagnosing microaspiration.^[6]^

Furthermore, compared to other markers and imaging studies, α-amylase and TUS are cheaper and easier to perform. However, the combination of TUS and α-amylase did not result in better VAP development than TUS alone. Our study had some limitations including its small sample size, the fact that not all patients received prophylactic antibiotics, and the fact that it was performed only in neurocritical patients. In addition, the imaging study was performed blindly by an expert, and we did not compare our results with those of another operator. This factor could probably have affected the results because there would be heterogeneity between each operator’s interpretations. However, adds to the knowledge of the relevance to detected lung changes with TUS before the appearance of VAP, by a no-radiation study bedside with high positive and negative predicted values and could be performed in less than 5 minutes, however, further studies are necessary to confirm these findings and see their scope.

## 5. Conclusions

An abnormal TUS pattern alone in the first 48 hours of orotracheal intubation is a significant predictor of VAP in neurocritical patients; however, we did not identify the utility of the combination of TA and TUS in predicting VAP.

## Author contributions

**Conceptualization:** Juan O. Galindo-Galindo, Jorge M. Llaca-Díaz.

**Data curation:** Juan O. Galindo-Galindo, Mario A. Ataxca-Gonzalez, Jorge M. Llaca-Díaz, Erick J. Rendón-Ramírez.

**Formal analysis:** Perla R. Colunga-Pedraza, Samantha P. Peña-Lozano, Erick J. Rendón-Ramírez.

**Investigation:** Roberto Mercado-Longoria, Perla R. Colunga-Pedraza, Jorge M. Llaca-Díaz, Erick J. Rendón-Ramírez.

**Methodology:** Roberto Mercado-Longoria, Juan O. Galindo-Galindo, Mario A. Ataxca-Gonzalez, Jorge M. Llaca-Díaz.

**Project administration:** Roberto Mercado-Longoria, Erick J. Rendón-Ramírez.

**Writing—original draft:** Perla R. Colunga-Pedraza, Erick J. Rendón-Ramírez, Erick J. Rendón-Ramírez.

**Writing—review and editing:** Perla R. Colunga-Pedraza, Samantha P. Peña-Lozano, Erick J. Rendón-Ramírez.
